# An intelligent decision support system for crop yield prediction using hybrid machine learning algorithms

**DOI:** 10.12688/f1000research.73009.1

**Published:** 2021-11-11

**Authors:** Kalaiarasi Sonai Muthu Anbananthen, Sridevi Subbiah, Deisy Chelliah, Prithika Sivakumar, Varsha Somasundaram, Kethaarini Harshana Velshankar, M.K.A.Ahamed Khan

**Affiliations:** 1 Faculty of Information Science Technology, Multimedia University, Bukit Beruang, Melaka, 75450, Malaysia; 2 Department of Information Technology,, Thiagarajar College of Engineering, Madurai, Tamil Nadu, India; 3 Faculty of Engineering, UCSI University, Kuala Lumpur, 56000, Malaysia

**Keywords:** Machine Learning, Prediction, Crop, Stacked Generalization, Random Forest, Regression

## Abstract

**Background**: In recent times, digitization is gaining importance in different domains of knowledge such as agriculture, medicine, recommendation platforms, the Internet of Things (IoT), and weather forecasting. In agriculture, crop yield estimation is essential for improving productivity and decision-making processes such as financial market forecasting, and addressing food security issues. The main objective of the article is to predict and improve the accuracy of crop yield forecasting using hybrid machine learning (ML) algorithms.

**Methods:** This article proposes hybrid ML algorithms that use specialized ensembling methods such as stacked generalization, gradient boosting, random forest, and least absolute shrinkage and selection operator (LASSO) regression. Stacked generalization is a new model which learns how to best combine the predictions from two or more models trained on the dataset. To demonstrate the applications of the proposed algorithm, aerial-intel datasets from the github data science repository are used.

**Results:** Based on the experimental results done on the agricultural data, the following observations have been made. The performance of the individual algorithm and hybrid ML algorithms are compared using cross-validation to identify the most promising performers for the agricultural dataset.  The accuracy of random forest regressor, gradient boosted tree regression, and stacked generalization ensemble methods are 87.71%, 86.98%, and 88.89% respectively.

**Conclusions: **The proposed stacked generalization ML algorithm statistically outperforms with an accuracy of 88.89% and hence demonstrates that the proposed approach is an effective algorithm for predicting crop yield. The system also gives fast and accurate responses to the farmers.

## Introduction

The tremendous increases in population and random climatic changes have laid down a great challenge to the agricultural sector in terms of the unavailability of food, productivity, and sustainability. Although farmers are skilled in the cultivation of crops, there is a huge gap between scientific and technological knowledge, and their availability in rural areas. One of the key challenges for a country's food security is climate change and its effects in the form of extreme weather events. The increase in temperature of 1-2.5 degrees Celsius forecast for 2030 is likely to have serious effects on crop yields ( Bhanumathi *et al.,* 2019) as it allows changes in photosynthesis, increases the respiration rate of plants, and affects pest populations.

One of the goals proposed to be achieved by 2030 is “no hunger” and the other goal is “promoting sustainable agriculture” ( Holzapfel and Brüntrup, 2017). Sustainable agriculture helps to empower small farmers, end poverty, improve the financial growth of the country, and to promote gender equality. The present scenario is alarming. To ensure sustainable access to nutritious food universally, countries would force continuous food production and agricultural practices ( Ramesh and Vardhan, 2015).

Timely and economic agricultural observance is essential to attain these goals. In this context, crop yield estimation is crucial for checking and making higher cognitive processes like crop insurance, money market foretelling, and addressing food security problems ( Patil and Shirdhonkar, 2017). With the drastic improvement in technology, the objective of the present study is to use the machine learning algorithms ( Medar *et al.,* 2019) and control systems to change the procedure and enhance the productivity ( Sriram *et al.,* 2019) of crops ( Zingade *et al.,* 2017).

Formerly, machine learning (ML) algorithms like linear regression and multiple linear regression have been used to make crop yield predictions ( Manjula and Djodiltachoumy, 2017). This article proposes improved ML algorithms that use specialized ensemble methods such as stacked generalization, gradient boosting, random forest, and least absolute shrinkage and selection operator (LASSO) regression. Our goal is to develop a web application in order to provide the farmers/users an approximation on how much amount of crop yield will be produced depending upon the given input and also find the relationship between yield (dependent variable) and other independent variables.

The remaining section of the article contains the literature survey, proposed method, results, discussion, conclusion, and recommendations for future work.

## Literature review

A convolutional neural network - recurrent neural network (CNN-RNN) framework for crop yield prediction was introduced by Saeed Khaki *et al.,* (2020). In this article, other models like random forest (RF), deep fully neural networks (DFNN), and LASSO algorithms were compared with CNN-RNN in predicting the corn and soybean yield. The forecasting was done throughout the Corn Belt within the United States for the years 2016, 2017, and 2018. The results were based on three categories, having soil, weather, and management as the attributes, and the accuracy for corn and soybean was 87.82% and 87.09% respectively.

To predict the crop yield, a random-forest classifier was used by Hajir Almahdi (2020) and Ramesh (2020). In their article, a graphical web-based interface was designed for a farmer to know the yield of crops beforehand cultivation. The dataset contains details about the crop production of Maharashtra where the study was conducted.

A backpropagation artificial neural network model was proposed by Meena and Singh (2013) for forecasting the crop yield. Unlike the fuzzy models, physical factors for yield forecasts were used. The annual forecast evaluation reports (AFER) are compared and have been reduced from 11.40% to 3.82%.

An empirical analysis for crop yield forecasting was done by Dharmaraja *et al.* (2020) as an attempt to focus on forecasting the yield of ‘bajra’ or the pearl millet crop through implementing appropriate statistical models such as regression and time-series models. Models like auto-regressive integrated moving average (ARIMA) and an ARIMA model with an exogenous variable (ARIMAX) were also used for prediction. The ARIMAX model produced the best outcome for 'bajra’ compared to the regression time series model.

A crop yield prediction using ML was proposed by Nishant *et al.* (2020). They used stacked regression for crop yield production, based on an additional factor of soil nutrients. Efficient neural network (ENeT), LASSO, and kernel ridge algorithms had minimal errors of 4%, 2%, and 1% respectively. A web page was used as an interface to display the predicted result.

Mobile based applications such as uzhavan ( https://apps.apple.com/in/app/uzhavan/id1405906962), Kisan ( https://apps.apple.com/in/app/kisan/id1297223018), and the agri app ( https://play.google.com/store/apps/details?id=com.criyagen&hl=en) provide facilities to the farmer for knowing the information about the scheme components, subsidy patterns, seeds and fertilizers. From the above literature, it is observed that the integration of an ML algorithm along with the web application or mobile application is missing. To address this issue, this article proposes a web page interface through which crop yield can be predicted with the applications of stacked generalization and random forest algorithms.

## Methods

Selecting appropriate data is a very important part of any machine learning algorithms or statistics. In the proposed system, Aerialintel datasets from the github data science repository were utilised to forecast crop yields ( Aerial Intelligence, 2017). Many researchers including Sriram Rakshith *et al.* (2019) and Jameshan (2017) have used this dataset and derived useful insights from it. It consists of two years’ winter wheat data for several counties in the United States of American for the years. 2013 and 2014, in total holding 26 attributes and over three hundred thousand records. The attributes mainly focus information about crop and climate data as outlined below.

**Figure 1.**
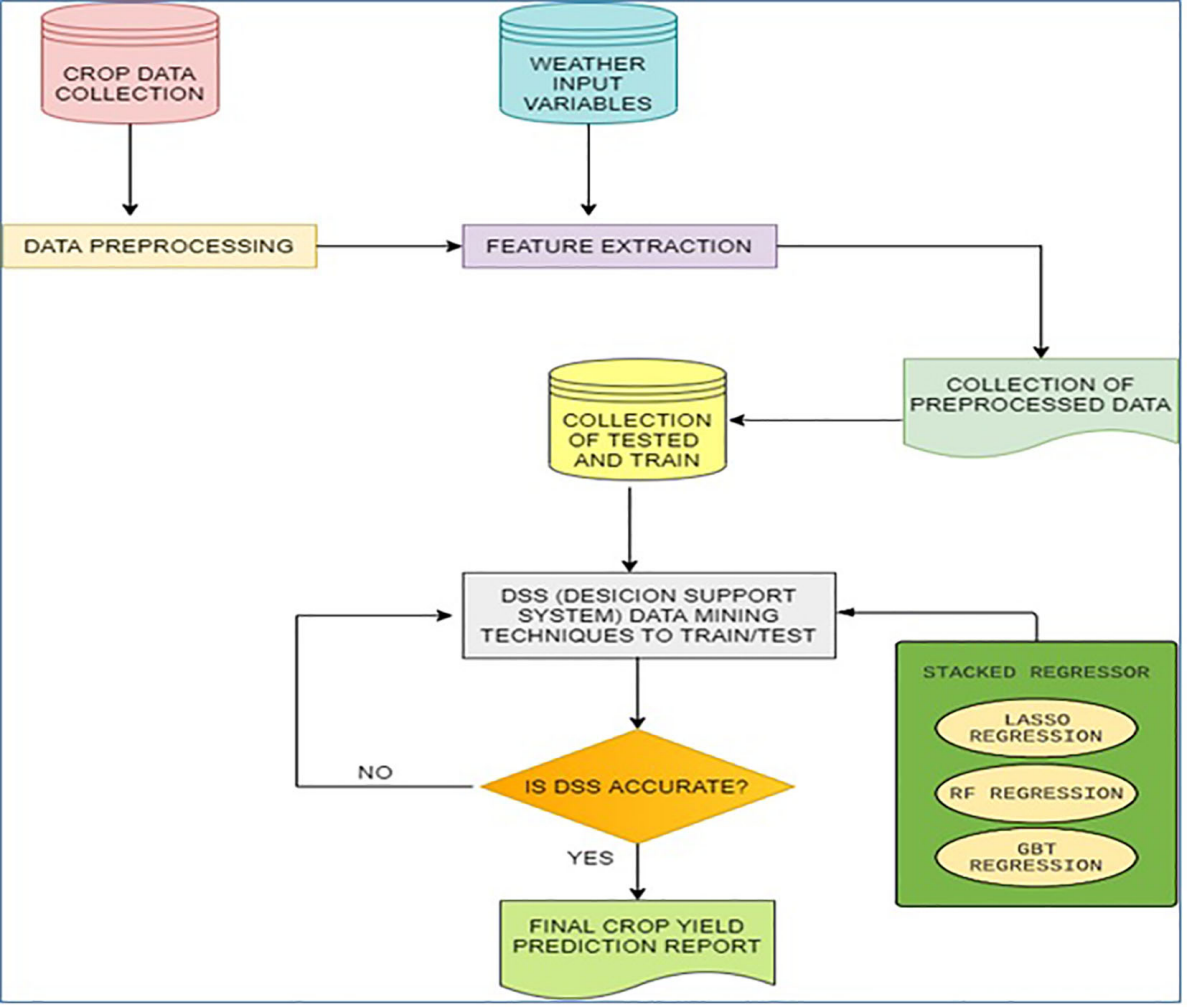
The framework for the proposed method.

The climatic parameters include precipitation, temperature, cloud cover, vapor pressure, and wet day frequency. The data in these files are geolocated to specific lat-longs and counties. The framework of the proposed work for this study using these datasets is shown in Figure 1. The framework contains the following modules: data preprocessing, feature extraction, and decision support system (DSS). DSS module includes predictions and performance evaluation.

Predictions can be done by stacked regressor and performance evaluation can be done by checking the accuracy of dss. The detailed explanation about performance evaluation has been discussed in the discussion section.

### Data preprocessing and feature extraction

In this phase, the collected dataset was explored, and data preprocessing techniques such as imputation of missing values and Haversine distance have been used. The details about the original dataset are shown in Table 1. Attributes precipIntensity, pressure, and visibility contain missing values. The number of missing values of the above attributes are 1, 254 and 30 respectively. Since the data are collected from different states in the United States, a global average cannot be used for imputing missing values. Therefore, the data from the same day and the closest neighboring location has been used to replace the null values by calculating the haversian distance between the two points. Basic statistics features like mean, variance, and quartiles values are computed for all the attributes. From which, it is found that the attribute “PrecipTypeIsOther” can be dropped as they hold no predictive power, since all the statistical values are around zero. Pairwise positive correlations between different features will aid the removal of features from models, as adding highly correlated features dilutes the model's predictive potential. Correlation coefficients have been estimated for all the possible combinations. From the correlation matrix, it has been observed that attributes like apparentemperaturemin, apparenttemperaturemax, and precipintensitymax etc have been removed since it is highly correlated with attributes like temperaturemax, temperaturemin, and precipAccumulation. The correlation between the attributes is given in the result section. After the removal of highly correlated attributes, the dataset contains the following attributes: latitude, longitude, precipAccumulation, temperaturemax, temperaturemin, ndvi,windspeed, country, state and date. Attributes such as State, and Date are removed because their inclusion would result in overfitting and a lack of generalization ( Gandhi *et al.,* 2016; Mythra *et al.,* 2018). Features like length of day and elevation plays an important role in crop yield prediction ( Nishant *et al.,* 2020). These features are the derived features, it is not available in the original dataset. Hence these two features of length of day and elevation were added in order to account for the amount of sunlight available to the plants at different locations. This can be done through astral package in python, version 3.8.8 ( https://www.python.org/downloads/release/python-388/). After data preprocessing and feature extraction, the dataset contains 12 features including derived attributes. These features are longitude, latitude, elevation, length_of_day, total_precipitation, minitemp, maxitemp, ndvi, windspeed, meantemp, stdtemp and yield. The results of the data preprocessing and feature extraction is shown in Figures 4– 6. The original dataset contains two years of winter wheat data for several countries in the United States of America for 2013 and 2014 together with python code for data preprocessing techniques such as correlation estimation and scatter matrix is uploaded in Github ( https://github.com/HangulAlien/intelligent-decision-support-system) ( HangulAlien, 2021).

**Table 1.** Original attributes along with the data type.

Attributes	Data type	Attributes	Data type
State	Char	precipAccumulation	Float
Latitude	Float	precipTypeIsRain	Int
Longitude	Float	precipTypeIsSnow	Int
Date	Char	precipTypeIsOther	Char
apparentTemperatureMax	Float	pressure	Float
apparentTemperatureMin	Float	temperatureMax	Float
cloudCover	Float	temperatureMin	Float
dewPoint	Float	visibility	Float
humidity	Float	windBearing	Int
precipIntensity	Float	windSpeed	Float
precipIntensityMax	Float	NDVI	Float
precipProbability	Float	DayInSeason	Int
country name	char	yield	Float

### Data partitioning

Based on Hajir Almahdi (2020) and Dharmaraja *et al.* (2020), the whole data set is divided into two parts: that is, 70% of the data set is used for training the model and 30% of the data is reserved for testing the model. In the 2013 wheat dataset, around 124,000 records were considered for training purpose and 53,000 records (containing the period from March to May 2014) were considered for testing purpose. In the 2014 wheat dataset, around 127,000 records were considered for training purpose and 54,000 records (containing the period from March to May 2015) were considered for testing purpose. While developing the machine learning model, both the datasets i.e., 2013 and 2014 datasets are combined.

A simple correlation study of the final featured data demonstrates that there was no strong linear correlation between the input features and the target output. However, some of them were linearly correlated to each other, which led to the conclusion that linear models such as linear regression could not be the best model for this dataset and problem. Hence, it was decided to execute many algorithms such as random forest (RF), stacked generalization, gradient boosted tree (GBT) regression, and LASSO regression algorithms ( Bhanu Kiran *et al.,* 2020). The efficiency of the model is tested using k-fold cross-validation ( Shah *et al.,* 2018; Champaneri *et al.,* 2020).

### Algorithms

In the proposed framework, the preprocessed dataset (contains 12 attributes), training and testing period is same for all the algorithms.

**Random forest (RF) regression:** The RF algorithm is a supervised learning model composed of multiple decision trees having the same nodes. It builds several decision trees and merges the decisions of several other decision trees to achieve a solution, which constitutes the mean of all these decision trees. The decision tree algorithm comprises traditional algorithms such as Iterative Dichotomiser (ID3), C 4.5 (which is a successor of ID3) and classification and regression tree (CART), etc. The performance of the algorithm can be measured by mean squared error (MSE).

$$\text{MSE}=\frac{1}{N}\sum_{i=1}^{N}{\left(f_{i}-y_{i}\right)}^{2}$$ (1)

where N is the number of records, $f_{i}$ is the value returned by the model, and $y_{i}$ is the actual value for the given data point.

**LASSO Regression:** LASSO regression is a form of linear regression that uses shrinkage. It performs both selections of variables and regularization in order to enhance accuracy. The LASSO model encourages simple, sparse models.

This precise form of regression is well-acceptable for models displaying excessive degrees of multi-collinearity or whilst one needs to automate certain components of model selection, like variable selection/parameter elimination.

$$L_{\text{lasso}}\left(\beta \right)=\sum_{i=1}^{n}{\left(y_{i}-\sum_{j}^{n}x_{\mathrm{ij}}\beta_{j}\right)}^{2}+\lambda \sum_{j=1}^{p}\left|\beta_{j}\right|$$ (2)

where $y_{i}$ is the outcome, $x_{\mathrm{ij}}$ is the covariate, λ is the amount of shrinkage and β is the regression coefficient.

**Gradient boosted tree (GBT) regression**: The GBT regression trees model is one of the most successful machine learning models for predictive study, which optimizes the result value in the successive steps in every iteration of the decision tree by adjusting the values of weights, or biases coefficients applied to the input variable. Gradient boosting involves three elements; namely, a loss function to be optimized, a weak learner to make predictions, and an additive model to add weak learners to minimize the loss function.

$$F_{m}\left(x\right)=F_{m-1}\left(x\right)+\sum_{j=1}^{J_{m}}\gamma_{\mathrm{jm}}1R_{\mathrm{jm}}\left(x\right),\gamma_{\mathrm{jm}}=\arg\;\min_{\gamma }\sum_{x_{i}\in R_{\mathrm{jm}}}^{\mathrm{Jm}}L\left(y_{i},F_{m-1}\left(x_{i}\right)+\gamma \right)$$ (3)

where $J_{m}$ is the number of terminal nodes in trees, $R_{\mathrm{jm}}$ is the region under study, $\gamma_{\mathrm{jm}}$ is the optimal value, and x is the training value.

**Figure 2.**
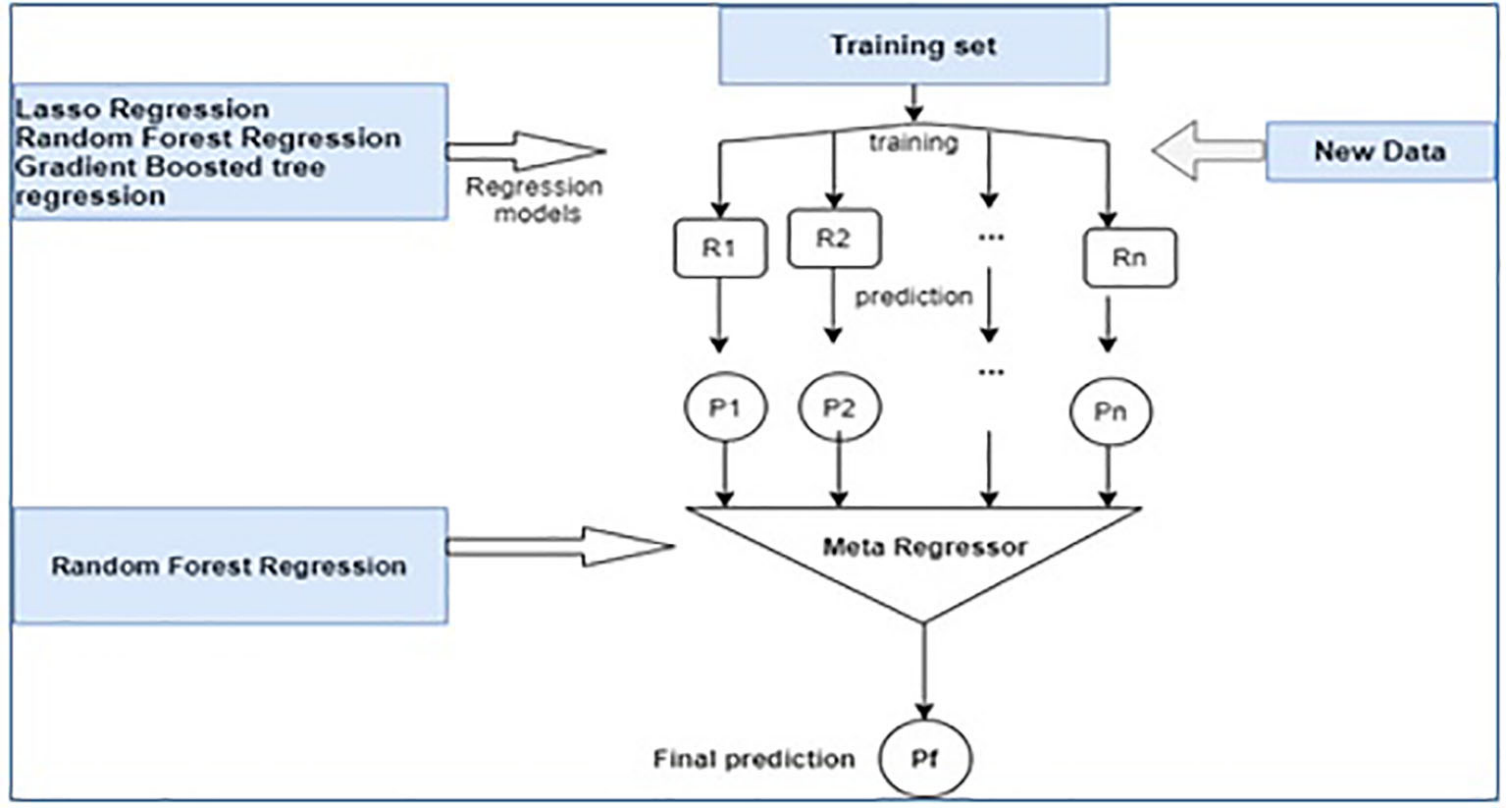
Stacking regressor.

**Stacked regression**: Stacking regressions is a method of combining multiple regressors to increase accuracy. The workflow of stacked regression is shown in Figure 2. It uses several meta-algorithms in order to learn how to combine the best predictions from two or more base algorithms. Here, by cross-validation and least square for non-negative values, the coefficient of the stack is found to give a result. It is found to be effective when compared with traditional ML algorithms and random forest. In the proposed work, the algorithms for random forest, LASSO regression, and GBT were used in the stacked regression. In Figure 2, R1, R2 … Rn represents the model which is generated after training. Based on the training model and testing data, the prediction models -P1,P2, … Pn is generated. The individual regression models are trained based on the same training set; then the meta-regressor is fitted based on the meta-features of the individual regression models in the ensemble learning. Meta-regression is a type of meta-analysis that customs regression analysis to combine, compare, and synthesize research findings from multiple experiments to provide a better response.

The Python integrated development environment (IDE) was utilised to find the machine learning solution for agricultural yield prediction using packages such as os, pickle, time, matplotlib, pandas, basemap, sklearn, numpy, and astral. Python pickle module is used for serializing and de-serializing a Python object structure. Pickle is used to “serializes” the object first before writing it to file. It is the way of converting a python object into a character stream. jsonify() is a helper method provided by Flask to properly return JSON data. Render_template is used to produce the output from a template file based on jinja2 engine. Render_template is typically imported directly from the flask package. Astral package is used to calculating the times of various aspects of the sun and phases of the moon.

**Figure 3.**
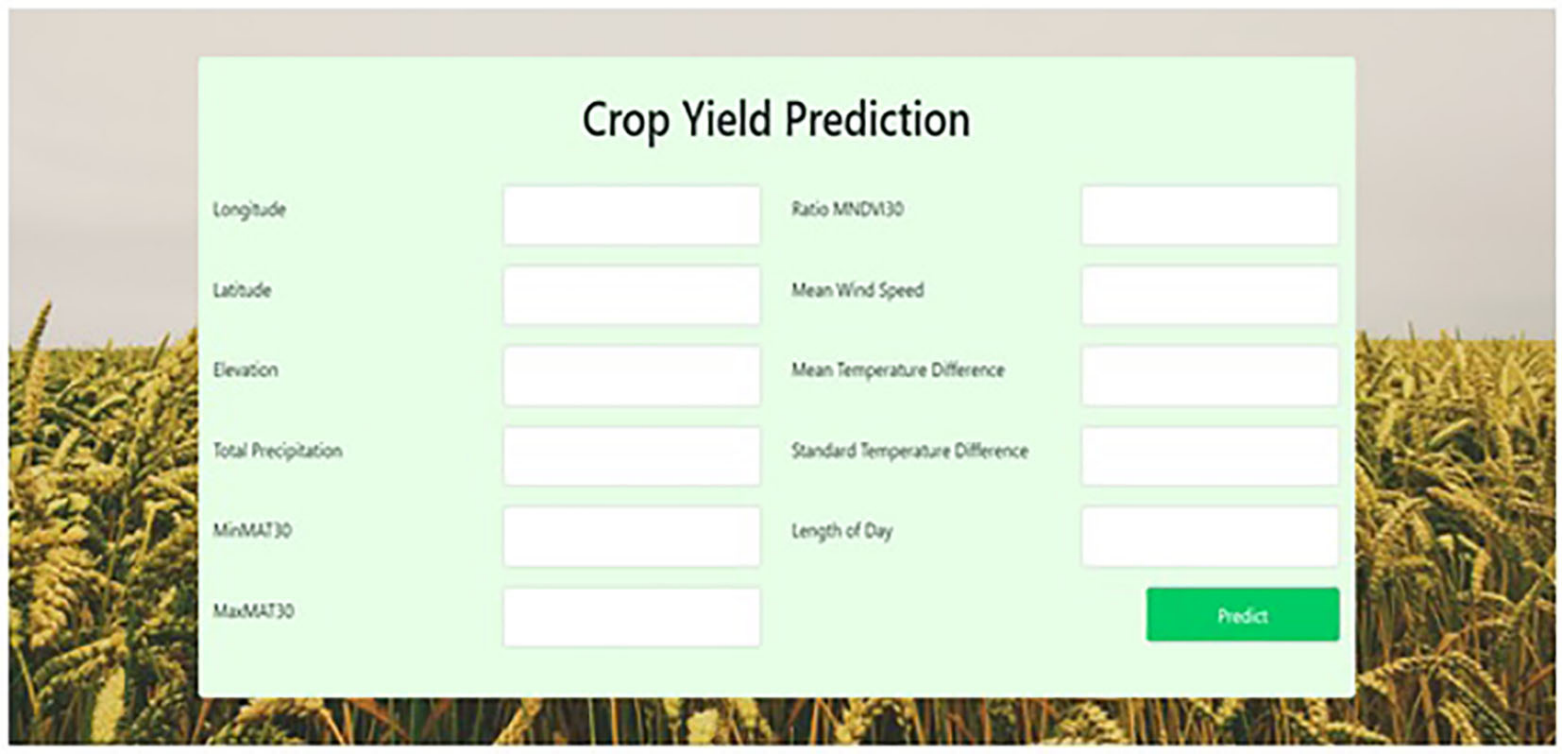
Web Interface to predict the crop yield.

The web-based model was deployed using flask, The flask framework's goal is to provide a graphical user interface for accessing information. In the proposed work, the best performing model i.e., stacked generalization is loaded in the flask framework to cross verify the performance or accuracy of the algorithm. When we provide inputs in the webpage, the stacked generalization model runs and provide the required output, i.e., yield prediction. The following input features: longitude, latitude, elevation, length_of_day, total_precipitation, minitemp, maxitemp, ndvi, windspeed, meantemp, and stdtemp are given in the web page to find the yield prediction. If the user enters the location details, wind speed and temperature details, they can obtain the yield prediction details. Around 100,000 records are considered for the testing purpose which includes combined data of 2013 and 2014 aerial intelligence (2017) datasets. Any novice users can access the webpage at any time from any location. The web interface is shown in Figure 3. The creation of the interactive page contains the following steps:

➢ Install the flask package available in python- version 3.8.8 ( https://www.python.org/downloads/release/python-388/).
➢ Create a HTML file to display the front-end design of the web page
➢ Create a python file that contains the following: generate a new route “/join” with “get and post” methods. Take the input from the web input box through request.form[<'name'>] . Perform the manipulations in the function and return the value as a JSON format to the web.
➢ Create a route “/” and return to html file from the function. Then run the python file and click on the link that it provides after running.
➢ The webpage takes the input from the web to flask and print the results back to the web page.

## Results

**Figure 4.**
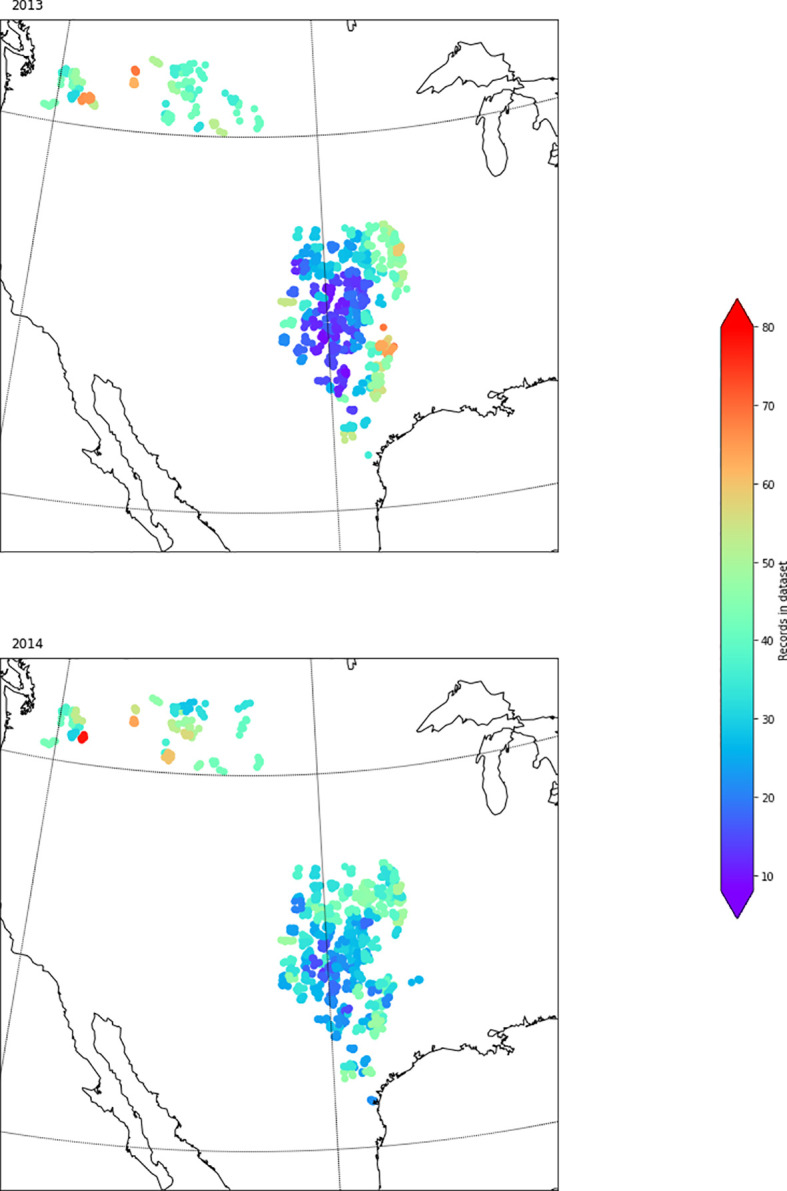
Geographical distribution of data in United States in 2013 and 2014.

**Figure 5.**
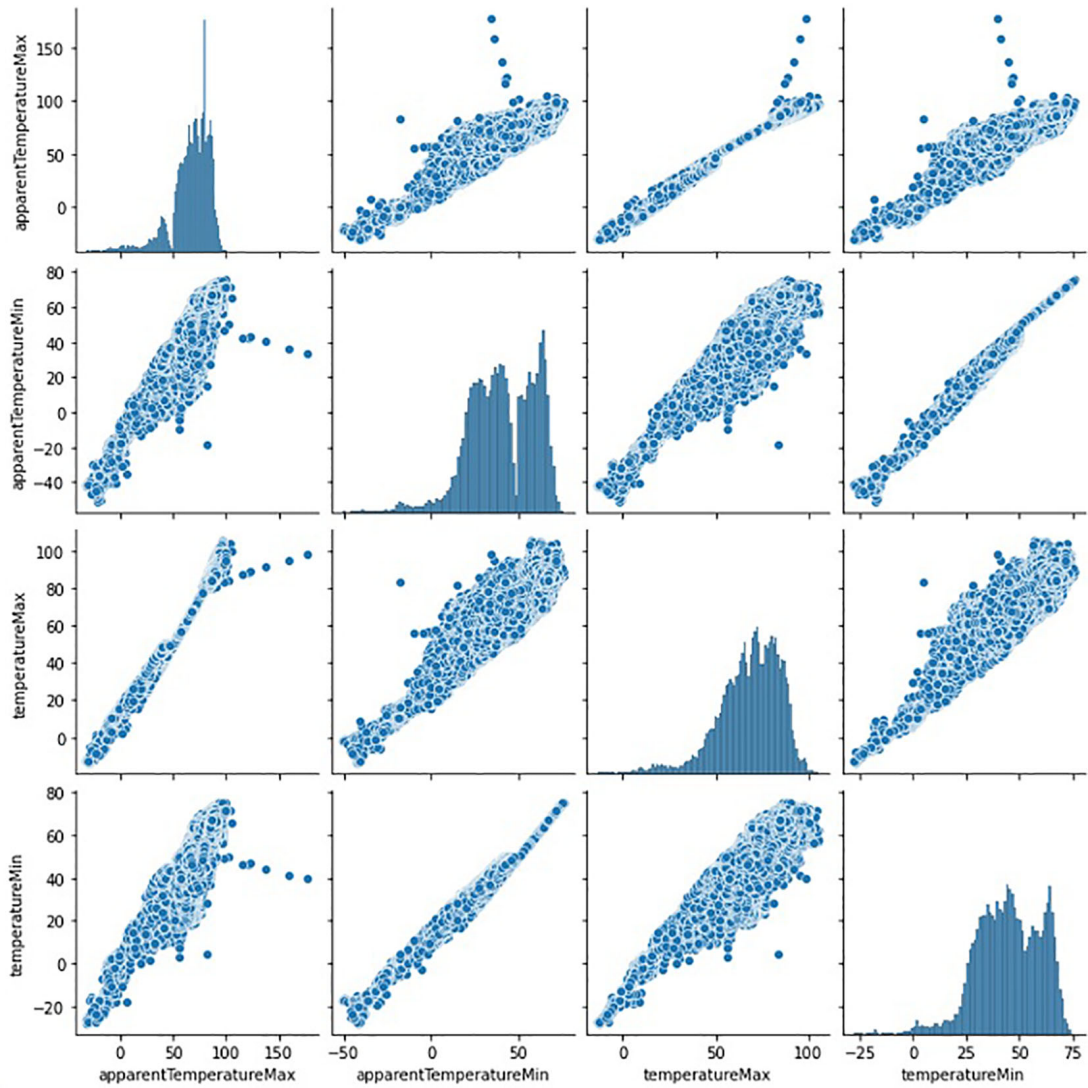
Scatter matrix for the 12 sample features.

**Figure 6.**
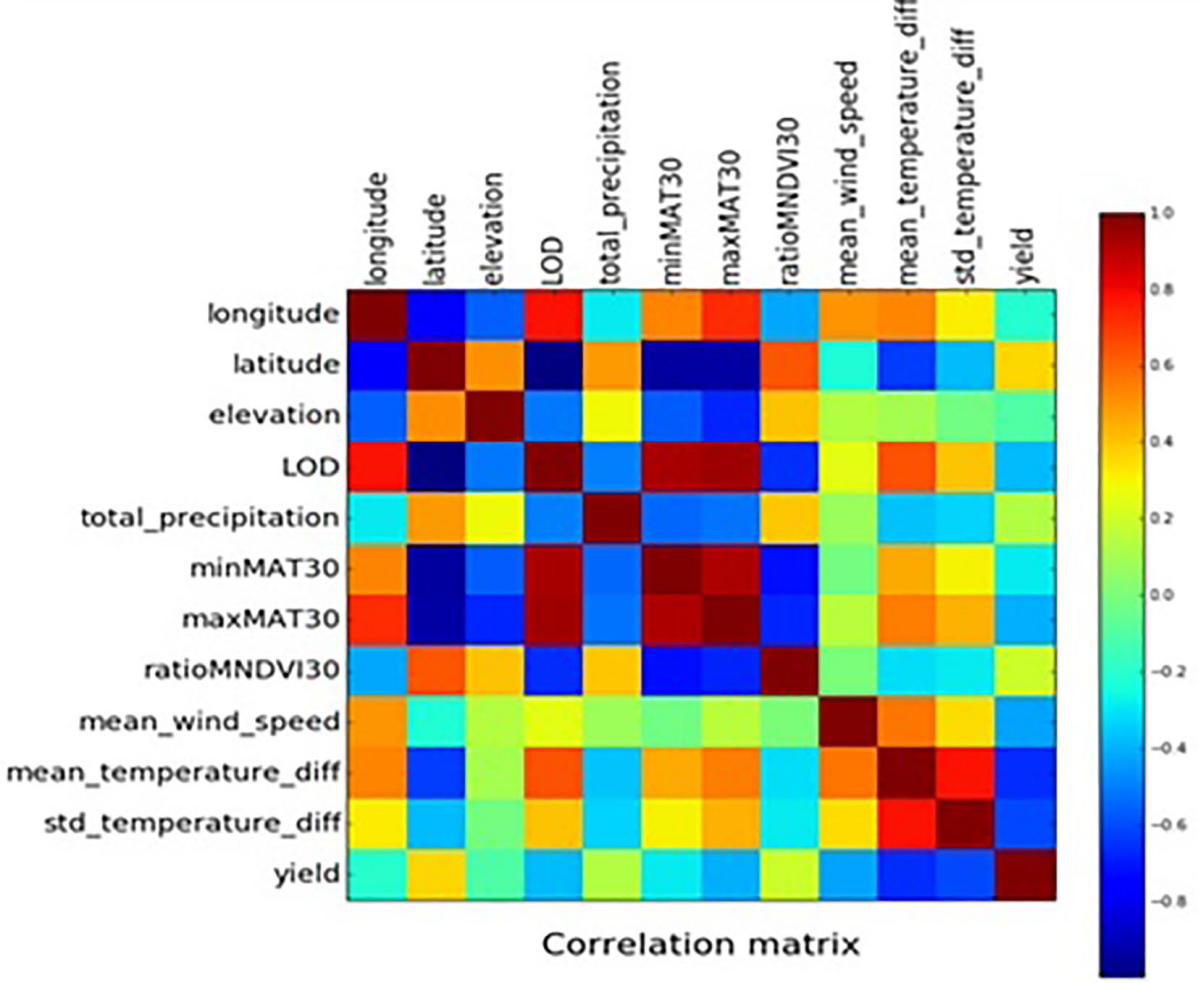
Correlation matrix in which highly correlated features are denoted in red and less correlated features are denoted in blue.

Figure 4 represents the geographical distribution of data corresponds to the years 2013 and 2014. This graph represents the yield of crops in the particular region based on the collected dataset. The red color denotes maximum yield while it decreases towards blue color. In the graph, the year representing the yield prediction and the number of records in the region are mentioned. The number of records mentioned in different colors starting from blue to red which resprents lowest to highest count of the record. Since the dataset is huge, here both scatter matrices and correlation matrix are used to find the correlation between two variables, is shown in Figures 5 and 6. To demonstrate the purpose of the scatter matrix, four features such as temperaruremin, tempertauremax, apparanttemperaturemin and apparanttemperaturemax is considered. As a result, a 4*4 scatter matrix has been formed and it is shown in Figure 5. In the matrix, diagonal value represents histogram of the above four attributes. Other than diagonal value represents, correlation between the attributes. For example, the first row represents the correlation between apparanttemperaturemax and the remaining attributes such as apparanttemperaturemin,tempertauremax and temperaruremin. From the first row, it is observed that apparanttemperaturemax is correlated with other three attributes, since the y value is increased if there is an increase in x value as well, as it contains very few outlier data. Similar to the first row, the correlation between the attributes can be taken from the second, third, and fourth rows. From the matrix, it is observed that all attributes are correlated with each other. The correlation between the 12 attributes is shown in Figure 6. In the correlation matrix, highly correlated features are denoted in red and less correlated features are denoted in blue. In the correlation matrix, the diagonal represents correlation of the univariate data. First row in the correlation matrix denotes how the attribute “longitude” is correlated with the other 11 attributes. From the first row of the figure, it is inferred that the attribute longitude is negatively correlated (blue color in Figure 6) with the attributes latitude, ratiomndvi30, and elevation. The attribute longitude has no correlation with the attributes total_precipitation and yield. The attribute longitude is positively correlated with the attributes minnat30, mean_wind_speed,std_temperature_diff, and mean_tempeaturediff. The attribute longitude is strongly correlated (red color in Figure 6) with the attributes LOD and maxmat30. Four regression-based algorithms were used to find the crop yield. They are random forest regression, gradient boosted tree regression, LASSO regression, and stacked generalization ensemble method. The relative efficiencies of these four models were compared using cross-validation as outlined in the methods section. The performance was measured by varying the hyper-parameter settings. In most of the cases, stacked generalization performed the best, followed by random forest, and gradient boosted tree regression. The overall comparison of the algorithms is shown in Table 2.

**Table 2.** Performance comparison of various machine learning algorithms.

Algorithm	Accuracy
Random forest regressor	87.71%
Stacked generalization	*88.89%*
Gradient boosted tree regression	86.98%
LASSO regression	42.00%

**Figure 7.**
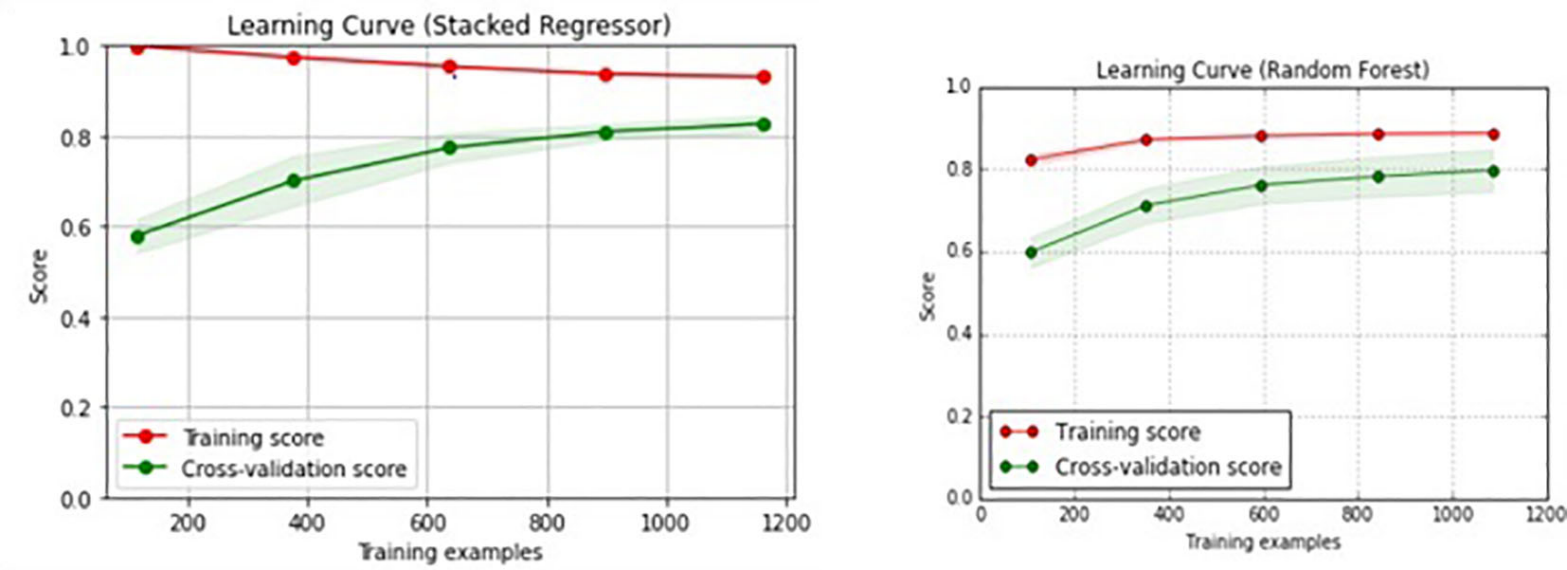
Learning curve of stacked regressor vs random forest.

When optimizing the parameters, the best pairs of hyper-parameters were found, from which the performance can be increased. The learning curve of the stacked regressor and random forest is shown in Figure 7. The proposed work is trained and tested. Based on the results obtained from the testing set, the comparison of the proposed algorithms has been done.

The proposed ensembling methods of stacked generalization, gradient boosting, random forest, and LASSO regression have been implemented using the same training dataset. Among these algorithms, random forest – 87.71% and stacked generalization – 88.89% yield slightly better accuracy than Kaur *et al.* (2020), who have implemented random forest, gradient boosted regression, nearest neighbor regression, and support vector machine with the polynomial kernel where the accuracy of the algorithms is 87.5%, 80.11%, 78%, and 34%, respectively.

## Discussion

**Figure 8.**
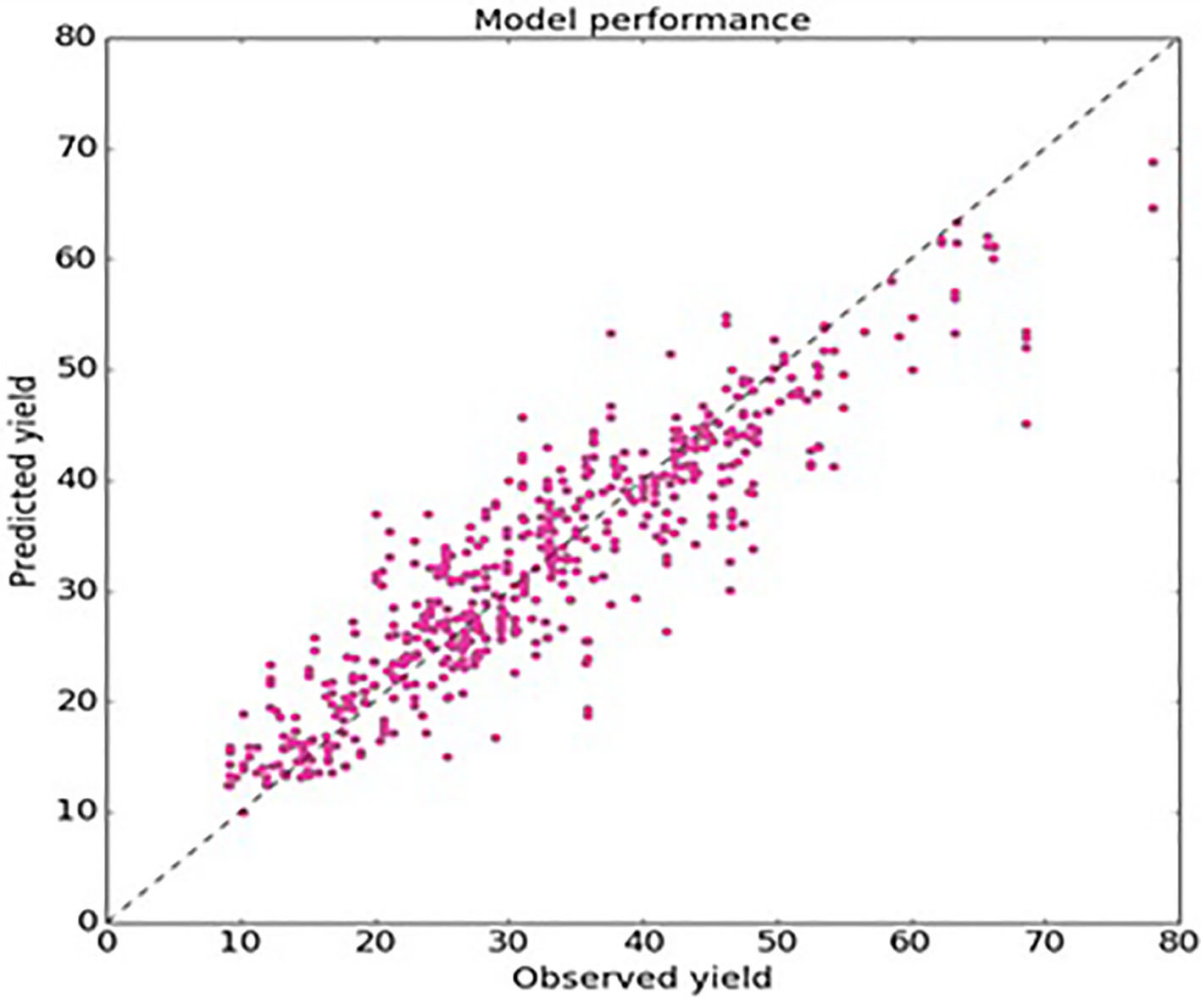
Performance of stacked generalization regressor.

The correlation matrix ( Figure 5) and the scatter matrix ( Figure 5) is used to find highly connected features ( Figure 6). Attributes like apparentemperaturemin, apparenttemperaturemax, and precipintensitymax have been removed since it is highly correlated with attributes like temperaturemax, temperaturemin, and precipAccumulation. Features like day length and elevation are added since they play an important role in crop yield prediction ( Nishant *et al.,* 2020). After data preprocessing techniques such as imputation of missing values, attribute elimination, and adding the new attributes, the dataset contains 12 attributes. The algorithms RF, stacked generalization, GBT regression, and LASSO regression is used to predict crop yield. The performance of these algorithms is shown in Table 2. The performance of each model is evaluated separately, and then the performance of the stacked regressor is evaluated. Among these algorithms, stacked regressor yield better results. The mean absolute percentage error is ~ 5%. Based on the experimental results outlined in the previous section, the following observations have been made. The accuracy of random forest regressor, gradient boosted tree regression, and stacked generalization ensemble methods are 87.71%, 86.98%, and 88.89 % respectively. The proposed stacked generalization ML algorithm statistically outperforms with an accuracy of 88.89% and hence demonstrates that the proposed approach is an effective algorithm. The learning curve (shown in Figure 7) for the training is above the validation score. This indicates the goodness of the random forest and stacked generalization model. The learning curve of the stacked generalization model ( Figure 8) showed little over-fitting but compared to other models, the overall accuracy and variance produce stronger results. The final model's R2 value is ~ 0.85 with a root mean square error (RMSE) of 5.2. The accuracy of the proposed algorithm is comparatively better than the existing work proposed by Kaur *et al. ,*2020. In the earlier literature ( Nishant *et al.,* 2020 and Medar *et al.,* 2019), yield prediction was done by accepting input parameters in the terminal and not in the web interface. The farmers don’t have knowledge and don’t know how to use the terminal. In the proposed framework, the above issue has been resolved by use of the web interface. In the literature ( Kaur *et al.,* 2020), they maily focused on latitude, longitude, temperature and humidity. They are not considering the derived attributes like elevation and the length_of_day. In the proposed work, including the above features totally 11 features longitude, latitude, elevation, length_of_day, total_precipitation, minitemp, maxitemp, ndvi, windspeed, meantemp, stdtemp are considered for predicting the crop yield. The testing dataset that supports to check the performance of the web interface. The interactive web interface is used to find the crop yield prediction by accepting the inputs from the user as shown in Figure 3. The limitation of the study is that the proposed work uses United States datasets by considering the crop yield for the year 2013 and 2014, where recent datasets have been considered for better understanding and checking the accuracy in the real time.

## Conclusions

Based on the climatic input parameters, the present experiment provided a demonstration of the possible use of four regression-based algorithms to predict crop yield. The algorithms are random forest regression, gradient boosted tree regression, LASSO regression, and stacked generalization ensemble method. In comparison of these algorithms, one concludes that the stacked ensemble model performed the best, followed by others for the given dataset.

Since this proposed system is a web-based system, input variables and modules can be easily changed as new features can be added based on their future needs. The system also gives fast and accurate responses to the farmers.

**Suggestion for future studies:** Our future work is to examine hybrid machine learning such as random forest, support vector machine, multiple regressor, logistic regressor and deep learning algorithms, such as deep convolution neural network (DCNN), and long short-term memory (LSTM) which might provide a fast and accurate solution to this problem. Future work will include considering the large recent datasets from different countries for predicting the crop yield in advance, leaf disease prediction, and predicting the quality of the fruits etc. and the results will be tested by the farmers and the agricultural experts.

## Data availability

### Underlying data

Zenodo: HangulAlien/intelligent-decision-support-system: Crop Prediction. https://doi.org/10.5281/zenodo.5533487 ( HangulAlien, 2021).

The project contains the following underlying data:

• Python file. (Contains code for Random forest, Gradient boosted tree regression, Lasso regression and stacked generalization).

Data are available under the terms of the Creative Commons Zero “No rights reserved” data waiver (CC0 1.0 Public domain dedication).

## Software availability

Source code available from: https://github.com/HangulAlien/intelligent-decision-support-system.

Archived source code at time of publication: https://doi.org/10.5281/zenodo.5533487.

License: Creative Commons Zero “No rights reserved” data waiver (CC0 1.0 Public domain dedication).
