# Supplementary material for: An intelligent decision support system for crop yield prediction using hybrid machine learning algorithms
Source: F1000Res. 2021 Nov 11;10:1143. [Version 1] doi: 10.12688/f1000research.73009.1 (PMC8689410; doi:10.12688/f1000research.73009.1)
Supplement: Peer review history

# Peer review: Reviewer response for version 1

**DOI:** 10.5256/f1000research.76627.r99829

**Published:** 2021-12-20

**Authors:** P. M. Durai Raj Vincent

**Affiliations:** 1 School of Information Technology and Engineering, Vellore Institute of Technology, Vellore, Tamil Nadu, India

This work on crop yield prediction is nicely presented with much clarity.

- The role of the crop yield prediction was explained well in the introduction section.
- Aerialintel datasets from the GitHub data science repository were utilized to forecast crop yields. The existing attributes, the reason for the elimination of certain attributes like "apparentemperaturemin", "apparenttemperaturemax", etc, and the reason for the inclusion of the attributes like length of day and elevation are explained in data reprocessing and feature extraction section.
- Figure 1 gives an overview of the entire work and it is easy to follow.
- The working of stacked generalization, gradient boosting, random forest, and least absolute shrinkage and selection operator (LASSO) regression for crop yield prediction was explained appropriately along with the equation.
- The author also explained the purpose and need of a web-based model. The web-based model was deployed using a flask.
- In the results and discussion section, the performance of the algorithms has been compared using accuracy and the learning curve. 

The following comment can be considered to further strengthen the work.

- The need for the current work was clearly mentioned in the Literature Review section by comparing with existing articles and existing mobile applications. However, many references are taken from conference proceedings, not from high-impact journals. The authors should consider references from high impact journal publications on crop yield prediction. For example, the following articles by this reviewer: Elavarasan *et al. *2018 ^1^, Elavarasan and Vincent 2021a ^2^ *, *Elavarasan and Vincent 2021b ^3^.

Finally, I conclude that the flow and contents are clear and the language is easy to understand. This article is suitable for indexing.

Is the work clearly and accurately presented and does it cite the current literature?

Yes

If applicable, is the statistical analysis and its interpretation appropriate?

Yes

Are all the source data underlying the results available to ensure full reproducibility?

Yes

Is the study design appropriate and is the work technically sound?

Yes

Are the conclusions drawn adequately supported by the results?

Yes

Are sufficient details of methods and analysis provided to allow replication by others?

Yes

Reviewer Expertise:

AI, ML, Deep Learning

I confirm that I have read this submission and believe that I have an appropriate level of expertise to confirm that it is of an acceptable scientific standard, however I have significant reservations, as outlined above.

## References

[1] : Forecasting yield by integrating agrarian factors and machine learning models: A survey. *Computers and Electronics in Agriculture* .2018;155: 10.1016/j.compag.2018.10.024 257-282 10.1016/j.compag.2018.10.024

[2] : Fuzzy deep learning-based crop yield prediction model for sustainable agronomical frameworks. *Neural Computing and Applications* .2021;33(20) : 10.1007/s00521-021-05950-7 13205-13224 10.1007/s00521-021-05950-7

[3] : A reinforced random forest model for enhanced crop yield prediction by integrating agrarian parameters. *Journal of Ambient Intelligence and Humanized Computing* .2021;12(11) : 10.1007/s12652-020-02752-y 10009-10022 10.1007/s12652-020-02752-y

# Peer review: Reviewer response for version 1

**DOI:** 10.5256/f1000research.76627.r99828

**Published:** 2021-12-20

**Authors:** Farrikh Alzami

**Affiliations:** 1 Faculty of Computer Science, University of Dian Nuswantoro, Semarang, Indonesia

The paper is well organized and easy to read to understand the content

 The paper used data which are collected from different states in the United States, a global average cannot be used for imputing missing values. Therefore, they applied the Haversian distance between the two points, for replacing the null values. 

 The authors stated that there is no strong linear correlation between the input features and the target output in the dataset. Hence, they decided to execute many algorithms such as random forest (RF), stacked generalization, gradient boosted tree (GBT) regression, and LASSO regression algorithms. And finally they proposed the Stacked regression model which is a method of combining multiple regressors and compared its performance with other models. 

 The authors compared the data set using Scatter matrix for the 12 sample features. They identified highly correlated features and less correlated features using heat map data visualization method.

 The authors concluded that the stacked ensemble model outperforms with an accuracy of 88.89%  than Random forest (87.71%) and Gradient Boosted tree (86.98%).

 The paper also stated the future works. Thus, I accept the paper without any modifications.

Is the work clearly and accurately presented and does it cite the current literature?

Yes

If applicable, is the statistical analysis and its interpretation appropriate?

Yes

Are all the source data underlying the results available to ensure full reproducibility?

Yes

Is the study design appropriate and is the work technically sound?

Yes

Are the conclusions drawn adequately supported by the results?

Yes

Are sufficient details of methods and analysis provided to allow replication by others?

Yes

Reviewer Expertise:

Data mining, machine learning, pattern recognition

I confirm that I have read this submission and believe that I have an appropriate level of expertise to confirm that it is of an acceptable scientific standard.

# Peer review: Reviewer response for version 1

**DOI:** 10.5256/f1000research.76627.r102293

**Published:** 2021-12-20

**Authors:** Millie Pant

**Affiliations:** 1 Department of Applied Science and Engineering, Department of Applied Science and Engineering, Roorkee, Uttarakhand, India

The authors have proposed an article entitled: An intelligent decision support system for crop yield prediction using hybrid machine learning algorithms.

 It is an interesting study and is relevant to the present scenario.

 I would suggest the authors to extend the review part a bit. Presently, it only discusses papers of 2020 and one paper of 2013. It will be good if the authors present a good range of review articles.

 In the opening sentence in the introduction, "increases" should be replaced with increase and "climatic" should be replaced with climate. Likewise there are other grammatical errors that may be corrected before submitting the final version.

 Maybe the authors can add a table mentioning the characteristics of the algorithms used in the paper.

Is the work clearly and accurately presented and does it cite the current literature?

Yes

If applicable, is the statistical analysis and its interpretation appropriate?

Yes

Are all the source data underlying the results available to ensure full reproducibility?

Yes

Is the study design appropriate and is the work technically sound?

Yes

Are the conclusions drawn adequately supported by the results?

Yes

Are sufficient details of methods and analysis provided to allow replication by others?

Yes

Reviewer Expertise:

Numerical optimization, artificial intelligence, data analysis

I confirm that I have read this submission and believe that I have an appropriate level of expertise to confirm that it is of an acceptable scientific standard.
